# 4143 HiREC Endowment: Building Models in Research Capacity for Infrastructure Sustainability and Productivity

**DOI:** 10.1017/cts.2020.214

**Published:** 2020-07-29

**Authors:** Lourdes E. Soto de Laurido, Walter R. Frontera, Aracelis Huertas

**Affiliations:** 1 University of Puerto Rico, Medical Sciences Campus

## Abstract

OBJECTIVES/GOALS: Improve infrastructure, resources, partnerships, and metrics to enhance the research environment for Hispanic researchers as a Minority Serving Institution. To support the research infrastructure in our Campus to encourage a research culture of sustainability and productivity. METHODS/STUDY POPULATION: Development of four research capacity-building models to enhance the pathway of junior researchers as independent researchers:1. MSc Phase I-Scholar Award 2 years in a Post Doctoral Master in CTR ; 2. Advanced CTR Award 1 year to support research infrastructure development in submitting a grant to NIH with the mentoring of a Visiting Endowed Chair; 3. Mini Infrastructure Research Award 1 year provides funds to increase research productivity; 4. Award on Excellence in CTR recognizes a faculty member with a distinguished research portfolio that support HiREC Career Coach and Mentoring approach. HiREC targets junior faculty, early and mid-career researchers from our two partners Schools. RESULTS/ANTICIPATED RESULTS: HiREC has been recognize as support for research infrastructure development. Since 2011, 10 MSc Phase I-Scholar Awards have been granted increasing the pool of trained Hispanics researchers in P. R., the Advanced CTR Award of $50,000 each, from March, 2019, was granted to 2 women researchers from the SoM and 2 Visiting Endowed Chair were accepted as candidates. The Mini Infrastructure Research Award, since 2017, supported the development of 2 Science labs, data analysis, 3 peer review publications and other research capacity building. Two researchers from the SoM were honored with the HiREC 2018 Award on Excellence in CTR heighten the institutional recognition of top researchers’ endeavors. DISCUSSION/SIGNIFICANCE OF IMPACT: It’s imperative to pursue specific strategies that lead to successful research capacity-building models. By acknowledging institutional research infrastructure needs, trendy scientific and technological knowledges and researchers’ needs, HiREC have been able to successfully accomplish its mission. CONFLICT OF INTEREST DESCRIPTION: Authors have no conflict of interest in this research.

